# Advice on better utilization of validation data to adjust odds ratios for differential exposure misclassification (recall bias)

**DOI:** 10.5271/sjweh.4226

**Published:** 2025-07-01

**Authors:** Igor Burstyn, George Luta

**Affiliations:** 1 Department of Environmental and Occupational Health, Dornsife School of Public Health, Drexel University, Philadelphia, PA 19104, USA. [E-mail: igor.burstyn@drexel.edu]; 2 Department of Biostatistics, Bioinformatics, and Biomathematics, Georgetown University Medical Center, Washington, DC 20057, USA.

We were delighted by the publication in your journal of the results of a validation study on self-reported night shift work by Vestergaard et al (1). Such exquisite validation studies that compare self-report to employment records are rare and sorely needed if we are to draw appropriate inferences from epidemiologic studies, both in characterization the degree of risk and – as recently argued by IARC – hazard identification (2). However, we have strong reasons to believe that the validation data which Vestergaard et al obtained could (and should) have been better used to “correct” odds ratios (OR) for differential exposure misclassifications.

[NB: Our use of the Excel spreadsheet of Lash et al (3) cited in Vestergaard et al (1) leads to the same “corrected” point estimate but a different, wider, 95% confidence interval (CI) 0.88–1.27. The corrected 95% CI reported in table 3 of (1) is obtained if we use rounded-up counts after adjustment. This is incorrect because expected counts “do not have to be integers”, as stated for the Excel spreadsheet that Vestergaard et al used. This illustrates the importance of the use of tools as intended and the unexpected impact on their results of apparently small changes to the input values for their calculations.]

**Table 3:** Summaries of posterior distributions after adjustment for differential exposure misclassification.

Parameter		Mean	Percentiles (%)		
			2.5	50.0	97.5
Odds ratio		0.98	0.30	0.97	1.71
Sensitivity					
	Controls	0.80	0.77	0.80	0.84
	Breast cancer patients	0.85	0.75	0.85	0.93
Specificity					
	Controls	0.84	0.82	0.84	0.86
	Breast cancer patients	0.81	0.75	0.82	0.87
Prevalence of exposure					
	Controls	0.15	0.12	0.15	0.18
	Breast cancer patients	0.14	0.05	0.15	0.22

First, we must note that quantitative bias analysis does not correct for exposure misclassification in general. In the case of using fixed values of sensitivities and specificities, it provides a corrected estimate only under the assumption that misclassification probabilities are known with absolute certainty. However, it is obvious from table 2 of Vestergaard et al (1) that misclassification probabilities are estimated with uncertainty. When there is uncertainty about sensitivities and specificities, the textbook they quote recommends (urges!) that probabilistic bias analysis should be carried out to account simultaneously for uncertainty in misclassification probabilities and random sampling errors (3). When this is done, probabilistic bias analysis does not guarantee the correction or adjustment for misclassification of exposure, but it merely produces a collection of alternative estimates via a Monte-Carlo simulation. An alternative adjustment approach for this case of uncertain exposure probabilities, which does involve theoretical assurance of correcting the OR for misclassification of exposure, is a Bayesian methodology (4, 5). Probabilistic bias analysis and Bayesian methods are not guaranteed to produce identical numerical results, and only Bayesian methods produce results that can be interpreted as distributions of true values given data, model and priors (6).

**Table 2:** Priors on misclassification probabilities derived from Vestergaard et al (**1**), **table 1** (see text for details).

Health status	Misclassification probabilities	Parameters of Beta distributions of misclassification probabilities			
		Shape 1 (α)	Shape 2 (β)	Mean (%)	Variance (%)
Breast cancer patients	Sensitivity	51	9	85	0.2
	Specificity	139	30	82.2	0.1
Controls	Sensitivity	375	91	80.5	0.03
	Specificity	1119	219	83.6	0.01

Second, it is known to be risky to adjust for exposure misclassification using fixed values of sensitivities and specificities if these are not known exactly (4). Small deviations from true misclassification probabilities can have a dramatic impact on the resulting adjustment. Thus, the corrected OR in Vestergaard et al (1) of 1.05 (95% CI 0.95– -1.16) is just one of many such adjusted estimates that is consistent with the presented validation data as we show below. Bayesian methods yet again come to the rescue here because they are designed to account for uncertainty in misclassification parameters by using prior probability distributions.

Third, we are puzzled by Vestergaard et al's choice of using the bootstrap to estimate distributions of sensitivities and specificities when there is a far simpler accepted approach to expressing uncertainty about proportions in quantitative bias analyses (Bayesian or probabilistic). When the validation study estimates a proportion k/N, the uncertainty about the true value of the proportion is typically expressed by using a Beta distribution, defined on [0,1] and is a conjugate prior of the Bernoulli distribution. For an observed proportion k/N, given that before performing the validation study we were completely ignorant about the value of the proportion, the Beta(α,β) distribution that captures this information has shape parameters α=k+1 and β=N-k+1, eg, see (7). We calculated these shape parameters for the misclassification probabilities from table 2 of Vestergaard et al (1) (this is partially reproduced in table 1) and presented them in our table 2, which also shows the corresponding means and variances.

**Table 1:** Validation data on misclassification probabilities presented in Vestergaard et al (1).

		Breast cancer patients				Controls		
		Payroll register				Payroll register		
		Ever-night shift work		Never night shift work		Ever-night shift work		Never night shift work
		Count		Count		Count		Count
Self-reported								
	Ever-night shift work	50		29		374		218
	Never night shift work	8		138		90		1118

Fourth, we observe that the Bayesian adjustment for differential exposure misclassification yields what may be considered as qualitatively different results compared to Vestergaard et al's adjustment of using fixed values. We followed the implementation from Singer et al (8). The Bayesian approach imposed no correlation between the misclassification parameters. We used a vague prior on the OR, null centered with 95% CI 0.02–50, as recommended for a sparse data problem (9). We also specified a uniform prior (0–1) on the exposure prevalence among controls. The Bayesian model converged and none of its diagnostics appear anomalous; implementation details that center around R (10) packages *rjags* (11) can be found in the supplementary material (www.sjweh.fi/article/4226) appendix A. Summaries of the posterior distributions are presented in table 3. The posterior OR adjusted for recall bias had a mean of 0.98, median of 0.97 and a credible interval of 0.30–1.71. As an added benefit, we have learned about the distributions of misclassification parameters and true prevalences, which can be used further if one is to update the study in question or use similar exposure assessment tools in a setting where similar exposure misclassification is suspected.

Lastly, we carried out our probabilistic bias analysis using the same Beta distributions as in table 2, assuming that the correlation of sensitivities and specificities is weak (ie, 0.1). Details of the implementation of probabilistic bias analysis using the R package episensor (12) are available in supplementary appendix B. The resulting simulated OR had a median of 1.00 and a 95% simulation interval of 0.48–1.31. Thus, Vestergaard et al (1) is an example of a study where using fixed values of misclassification probabilities leads to a rather different estimate of 1.05 (and corresponding 95% CI 0.95–1.16) compared to both probabilistic bias analysis and Bayesian adjustment method that use the same validation data. Distributions of OR obtained after probabilistic and Bayesian adjustments are illustrated in figure 1, which shows that the Bayesian method (in red) favors lower true values of the OR compared to the probabilistic one (in gray).

**Figure 1:**
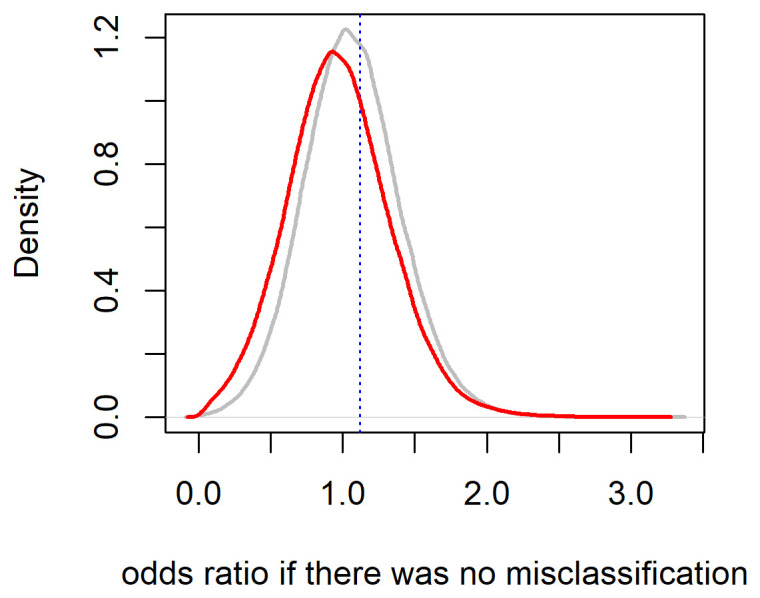
Distribution of odds ratios adjusted for misclassification using Bayesian method (red) and probabilistic bias analysis (grey); the location of naïve point estimate is denoted by a blue dotted line

When faced with numerically different results of adjustment for exposure misclassification, we advise our colleagues to rely on the results that arise from the more theoretically justified methodology. In the case of adjustment from Vestergaard et al (1), we think that the Bayesian results are more defensible, yielding an adjusted OR centered around 1.0 (95% credible interval 0.3–1.7). This result appears to us to be a rather more convincing estimate for the association of breast cancer with report of ever having worked night shifts than Vestergaard et al's “corrected” estimate. We urge epidemiologists who collect precious validation data to collaborate with statisticians who can help them fully utilize it, arriving at more defensible effect estimates and, ultimately, better risk assessments.

## Supplementary material

Supplementary material
