# Supplementary material for: Advice on better utilization of validation data to adjust odds ratios for differential exposure misclassification (recall bias)
Source: Scand J Work Environ Health. 2025 Jun 26;51(4):344–6. doi: 10.5271/sjweh.4226 (PMC12282599; doi:10.5271/sjweh.4226)
Supplement: Supplementary material [file SJWEH-51-344-S001.pdf]

Advice on better utilization of validation data to adjust odds ratios for differential exposure misclassification (recall bias)[1]

Igor Burstyn, PhD,[2] George Luta, PhD

1. Supplementary appendices
2. Correspondence to: Department of Environmental and Occupational Health, Dornsife School of Public Health, Drexel University, Philadelphia, PA 19104, USA. [E-mail: igor.burstyn@drexel.edu]

Appendix A: Bayesian adjustment for exposure misclassification:

```
####################################################
#Bayesian differential exposure misclassification
#recall bias adjustment
#two-by-two table
####################################################

install.packages(c("rje","rjags","MCMCvis")) #do only ONCE for a R installation, not every
time you open R

#load required R packages every time you open R
require("rje")
require("rjags")
require("MCMCvis")

set.seed(31415926) #fix random number seed to aid reproducibility

#A JAGS model.
#lor ~ dnorm(0,0.25) #prior on log-OR vague and null-centered approx (N(mu=0,var=4)) =>
95% range 0.02 to 50
                    ##Greenland's choice for data augmentation and control for
sparse data bias
#lor ~ dnorm(0,1)    #prior on log-OR vague and null-centered approx (N(mu=0,var=4)) =>
95% range 0.14 to 7

genmod.string <- "model{
#data
x0 ~ dbin(p0, n0)
x1 ~ dbin(p1, n1)
#model of observed probabilities of exposure p0=controls, p1=cases
```

```
#true exposure probability of cases = r1, among controls = r0
p0 <- r0*SN0 + (1-r0)*(1-SP0)
p1 <- r1*SN1 + (1-r1)*(1-SP1)
r1 <- (OR*r0)/(1-r0+OR*r0)
#priors
r0  ~ dbeta(aa,bb) #can make informative as per prior elicitation but flat initially as
beta(1,1)
lor ~ dnorm(0,0.25) #prior on log-OR vague and null-centered approx (N(mu=0,var=4)) =>
95% range 0.02 to 50
#misclassification among referents
SN0 ~ dbeta(a.sn0, b.sn0)
SP0 ~ dbeta(a.sp0, b.sp0)
#misclassification among cases
SN1 ~ dbeta(a.sn1, b.sn1)
SP1 ~ dbeta(a.sp1, b.sp1)
#calculations of parameters of interest = true odds ratio
OR <- exp(lor)
 }"


#in data, specify two-by-two table
#x0 and x1 are numbers of exposed referents and cases, respectively
#n0 and n1 are numbers of referents and cases, respectively
##data are from Hoppin et al as determined during 11/8/2024 workshop
# a* and b* are the shapes 1 and 2 of Beta distributions
#sn0 = sensitivity among referents/controls
#sp0= specificity among referents/controls
#sn1 = sensitivity among cases
#sp1= specificity among cases

mod <- jags.model(textConnection(genmod.string),
data=list(x0=1560, x1=1695, n0=6000, n1=6000,
a.sn0=375, b.sn0=91,
a.sp0= 1119, b.sp0=219,
a.sn1=51, b.sn1=9,
a.sp1=139, b.sp1=30,
aa=1, bb=1),
n.chains=3)

update(mod, 100000)   #burn-in

opt.JAGS <- coda.samples(mod, n.iter=50000,
        variable.names=c("SN0","SP0", "SN1", "SP1", "OR", "lor", "r0", "r1"))
```

```
#What comes out: samples from posterior distributions: true values given data, model,
and priors

MCMCsummary(opt.JAGS)
MCMCtrace(opt.JAGS, params=c("OR", "lor"), pdf=F)
MCMCtrace(opt.JAGS, params=c("SN0", "SN1"), pdf=F)
MCMCtrace(opt.JAGS, params=c("SP0", "SP1"), pdf=F)
MCMCtrace(opt.JAGS, params=c("r0", "r1"), pdf=F)

#save CODA to process
#raw.MCMC <- MCMCchains(opt.JAGS)
#histogram of posterior of "OR" for the Figure
#hist(raw.MCMC[,"OR"])


####THE END####
```

Appendix B: Probabilistic Bias Analysis:

```
#adjust for recall bias estimated in
#Vestergaard et al.(2024).
#"Validity of self-reported night shift work among women with and without breast cancer."
#Scand J Work Environ Health 50(3): 152-157.



######Lash, Fox, et al.
install.packages("episensr") #do only once to install library that has function probsens()
library(episensr)# do every time you call function
set.seed(31415926) # make reproducible

#####################################
#contingency table
#####################################
table<-matrix(c(1695,4305,1560,4440),
dimnames=list(c("case","control"),c("exposed","unexposed")),nrow=2,byrow=TRUE)
table

#########################################################################
#########
#beta priors on SN and SP
```

```
#######################################################################
#########

probsens(table,
type = "exposure",
reps = 20000,

#sensitivities
  #cases
seca.parms = list("beta", c(51, 9)),
 #controls
seexp.parms = list("beta", c(375, 91)),

#specificities
 #cases
spca.parms = list("beta", c(139, 30)),
 #controls
spexp.parms = list("beta", c(1119, 219)),
corr.se=.1, corr.sp=.1)


####THE END####
```
